# Changes in prevalence of tobacco use and the factors that may affect use among Uganda youth: the Global Youth Tobacco Survey (GYTS) 2007-2011

**DOI:** 10.11604/pamj.2016.25.152.9991

**Published:** 2016-11-14

**Authors:** Daniel Kadobera, Martine Chaussard, Kyung Ah Lee, Nicholas Ayebazibwe, Sheila Ndyanabangi

**Affiliations:** 1 Office of Mental Health and Substance Abuse, Ministry of Health, Government of Uganda; 2 Global Health, American Cancer Society (ACS); 3 Office of Smoking and Health, U.S. Centers for Disease Control and Prevention; 4 African Field Epidemiology Network (AFENET)

**Keywords:** Tobacco use, youth, GTSS, GYTS, tobacco control, FCTC, prevalence

## Abstract

**Introduction:**

To assess changes from 2007 to 2011 in the prevalence of tobacco use and tobacco-related indicators in Uganda by examining results from the Global Youth Tobacco Survey (GYTS).

**Methods:**

Both the 2007 (n=2,251) and 2011 (n=2,026) Uganda GYTS were conducted among students in primary seven, secondary one, two, and three. A two-stage cluster sample design was used to generate a representative sample of students for the surveys. Stata 12 software was used to provide weighted prevalence estimates and logistic regression models were developed to examine the relationship between factors that influence tobacco use and current tobacco use.

**Results:**

The percentage of students who had ever smoked a cigarette, even just one or two puffs, declined from 15.6% in 2007 to 10.9% in 2011 (p=0.03). From 2007 to 2011, neither the percentage of current use of any tobacco (16.6% to 17.3%, p=0.75), nor the percentage of current (past 30 day) cigarette smoking (5.5% to 4.8%,p=0.59) changed significantly. Following adjustment, having parents [Adjusted Odds Ratio (AOR):1.9, 95% Confidence Interval (CI):1.3-2.8] and friends [AOR 2.5, 95% CI: 1.5-4.0)] who smoke, and having seen tobacco advertisements in print media [AOR 1.8(1.3-2.4)], were associated with greater odds of current tobacco use among students in 2007.Having parents who smoke [AOR;1.8, 95% CI: 1.1-3.0] was associated with greater odds of current tobacco use among students in 2011.

**Conclusion:**

From 2007 to 2011, no significant change occurred in the prevalence of current tobacco use or cigarette smoking among youth in Uganda. These findings underscore the importance of implementing effective population-level public health interventions, as outlined in the articles of the World Health Organization’s Framework Convention on Tobacco Control, to prevent and reduce the use of tobacco among youth in Uganda.

## Introduction

Non communicable diseases are the leading causes of death globally, killing more people each year than all other causes combined; tobacco use represents the leading cause of non-communicable diseases worldwide [1]. The consumption of tobacco represents a major risk factor for morbidity and mortality from a variety of major health outcomes, including various cancers, stroke, chronic obstructive pulmonary disease, and other respiratory and cardiovascular diseases, as well as adverse reproductive health outcomes and Sudden Infant Death Syndrome (SIDS) [2, 3]. Annual tobacco-related deaths have been estimated at more than 8 million globally, and these deaths impose an annual economic cost of hundreds of billions of U.S. dollars [1]. Accordingly, implementing effective ways to prevent cigarette smoking and other forms of tobacco use can yield important public health benefits, particularly in low and middle income countries.

The World Health Organization (WHO) has identified six evidence-based measures to reduce tobacco use, as outlined in the Framework Convention on Tobacco Control (FCTC) [4], which provides the driving force and blueprint for the global response to the tobacco epidemic. Known as “MPOWER,” these measures are as follows: Monitor tobacco use and prevention policies, Protect people from tobacco smoke, Offer help to quit tobacco use, Warn people about the dangers of tobacco, Enforce bans on tobacco advertising, promotion and sponsorship, and Raise taxes on tobacco. In 2015, Uganda passed an FCTC compliant law with all the major clauses related to MPOWER, and implementation of the law is currently in process [5].

To date, Uganda has carried out three rounds (2002, 2007 and 2011) of the Global Youth Tobacco Survey (GYTS), as part of the Global Tobacco Surveillance System (GTSS), which provides critical information on tobacco use and key tobacco control indicators for policy makers [6]. To date, no study has assessed key indicators from the Uganda GYTS to determine shifts in tobacco use, as well as factors that may be influencing use, over time. To address this gap, we assessed the prevalence of tobacco use between 2007 and 2011 from the Uganda GYTS, and explored changes in key factors that may have influenced youth tobacco use over this period. The 2002 GYTS was not included in the present analysis, as it was conducted in just two regions of the country, and thus, was not nationally representative. It’s expected that the findings from this study may help inform the implementation and evaluation of tobacco-related interventions, as outlined in MPOWER, among youth in Uganda.

## Methods

### Data source

The aim of the GTSS is to provide country specific data to design, implement, and evaluate tobacco control interventions. A component of GTSS, the GYTS, is a school-based survey that uses a standardized methodology to select sample frames, schools and classes. The questionnaire, implementation of fieldwork, data management, and processing of the data are all standardized [7]. A more comprehensive description of the overall GYTS purpose and the methods used is available elsewhere [7, 8]. In brief, the GYTS is self-administered in classrooms, and the voluntary nature of participation and anonymity of the students are maintained throughout the survey process. A core set of questions, consistent across all GYTS implementing countries, is coupled with a set of optional, country-specific questions.

In Uganda, both the 2011 and 2007 GYTS used a two-stage cluster sample design to produce a nationally representative sample of students in grade 7 of primary and grades 1 to 3 of secondary education who were aged 13-15 years. A total of 2,251 and 2,026 students between the ages of 13-15 years were surveyed in 2007 and 2011 respectively. The overall student response rate was 81.2% in 2007 and 83.3% in 2011.

### Measures

The data presented in this study are based on responses to questions in two areas: tobacco use and factors that may influence use. Tobacco use was measured by: current (past 30 day) use of any tobacco cigarettes smoking; use of smoked tobacco other than cigarettes; use of smokeless tobacco. Survey questions about factors that may influence tobacco use included whether one or both parents smoke; having friends who smoke; holding the belief that tobacco smoke is harmful to others; having seen pro tobacco ads on billboards (in the last 30 days); having seen pro-tobacco ads in newspapers or magazines (last 30 days); possessing an item (such as a T-shirt or pen) with a cigarette logo or brand name on it; having been offered a free cigarette by a tobacco industry representative (last 30 days); having been taught in school the danger of smoking tobacco; and having been taught in school the effects of smoking tobacco on health.

### Analysis

A weighting factor was applied to each student record to adjust for non-response and for variations in the probability of selection by school, class, and student levels [9]. Post-stratification calibration by grade and sex was applied to weight the sample to the population of school children in the selected grades at each sample site. Prevalence estimates and 95% confidence intervals (CIs) were calculated [9]. A two-sample t-test was used to assess the difference between years of the sample (2007 and 2011) (α=0.05). Additionally, univariate and multivariate logistic regression models were developed to examine the correlation between factors that may influence tobacco use and the prevalence of current tobacco use.); adjusted odd ratios were calculated with 95% confidence intervals. Only variables found to be significantly associated with current tobacco use in the univariate analyses were included in the multivariate models. All analyses were conducted using STATA version 12.

## Results

### Tobacco use

The estimated percentage of students who had ever smoked a cigarette, even one or two puffs decreased from 15.6% (12.9-18.3%) in 2007 to 10.9% (7.4-14.3%) in 2011 (p<0.05). The overall percentage of current tobacco use of any type did not change significantly from 2007 (16.6% [95% CI: 14.2-19.0%]) to 2011 (17.3% [95% CI: 14.1-20.4%]) (p=0.751) (Table 1). Similarly, no statistically significant change was observed between 2007 and 2011 for past 30 day use of other forms of smoked tobacco (8.8% to 9.7%, p=0.5438) or smokeless tobacco (9.4% to 10.0%, p=0.6589). In 2007, 11.5% (8.7-14.3%) of the students had at least one parent who smoked, as compared to 10.7% (6.7-14.6%) in 2011 (Table 2). The overall estimate for having friends who smoked was 2.3% (1.8-2.9%) and 2.5% (1.2-3.8%) in 2007 and 2011, respectively.

**Table 1:** Percentage of students aged 13-15 years by tobacco use status, Global Youth Tobacco Survey (GYTS) Uganda, 2007 and 2011

	2007	2011	
Tobacco use status	% (95% CI)	% (95% CI)	p-value
Current tobacco use (last 30 days)			
Yes	16.6(14.2-19.0)	17.3(14.1-20.4)	0.751
Ever smoked a cigarette, even one or two puffs			
Yes	15.6(12.9-18.3)	10.9(7.4-14.3)	0.0302^+^
Currently smoked cigarettes (last 30 days)			
Yes	5.5(4.0-6.9)	4.8(2.9-6.8)	0.5856
Currently used other forms of smoked tobacco (last 30 days)			
Yes	8.8(7.0-10.6)	9.7(7.4-11.8)	0.5438
Currently used smokeless (last 30 days)			
Yes	9.4(8.0-10.7)	10.0(7.4-12.7)	0.6589

Note: values with ^+^indicate statistical significance (p < 0.05)

**Table 2:** Exposure to risk factors for tobacco use, and correlation with current tobacco use, among students aged 13-15 years; Global Youth Tobacco Survey (GYTS) Uganda, 2007 and 2011

Risk factor for current tobacco use	2007		2011	
	% (95% CI)	AOR^+^ (95% CI)	% (95% CI)	AOR^+^(95% CI)
**Sex**				
Male	49.4(43.3-55.4)	NA	43.8(38.4-49.1)	NA
Female	50.6(44.6-56.7)		56.2(50.8-61.6)	
**Age**				
13	21.0(16.6-25.3)	NA	22.4(17.4-27.4)	NA
14	33.6(31.7-39.4)		40.8(37.0-44.5)	
15	43.5(37.3-49.6)		36.9(31.1-42.5)	
**Parents Smoke**	11.5(8.7-14.3)	**1.9 (1.3-2.8)**	10.7(6.7-14.6)	**1.8 (1.1-3.0)**
**Friends Smoke**	2.3(1.8-2.9)	**2.5 (1.5-4.0)**	2.5(1.2-3.8)	1.4 (0.5-3.9)
**Taught smoking effects at school**		NA		NA
	69.4(66.0-72.9)	NA	75.0(63.6-86.5)	NA
**Taught smoking danger signs at school**	70.5(67.3-73.8)		73.8(64.3-83.3)	
**Has an object with a tobacco logo**	12.3(10.4-14.1)	1.6 (1.0-2.8)	11.3(8.9-13.6)	1.3 (0.6-2.6)
**Seen tobacco ads on billboards**	54.7(51.1-58.3)	1.0 (0.6-1.5)	52.1(43.0-61.1)	1.2 (0.8-1.7)
**Seen tobacco ads in print media**	48.3(45.8-50.9)	**1.8 (1.3-2.4)**	46.5(36.9-56.1)	NA
**Been offered free cigarettes**	10.3(8.1-12.6)	1.3 (0.8-2.1)	10.7(7.2-14.1)	1.0 (0.4-2.5)

Abbreviations: AOR: Adjusted odds ratios; CI: Confidence interval NA: Not applicable - variables without statistical significance in univariate analysis were excluded from the multivariate analysis.

+ Statistically significant odds ratios noted in bold. Only variables for which the odds ratio did not include 1.0 in the confidence intervals and which were therefore statistically significantly related to tobacco use in the univariate analyses were included in the adjusted model. Independent variables are dichotomous (1=Yes and reference =No).

Following multivariate adjustment, students who have parents who smoke had approximately twice the odds of being current tobacco users [AOR: 1.9 (1.3'>AOR: 1.9 (1.3-2.8) for 2007 and AOR 1.8 (1.1-3.0) for 2011] (Table 2). Students who have friends who smoke had over twice the odds (AOR: 2.5; 95%: CI 1.2-3.8) of being current smokers in 2007; in contrast, there was no statistically significant association observed for 2011. Students who reported having seen tobacco advertisements in print media had higher odds (AOR: 1.8 (95% CI: 1.3-1.24) of being current tobacco users in 2007, while in 2011, no statistically significant association was observed.

## Discussion

The findings in this study show that the overall prevalence of current cigarette smoking did not change significantly among school children aged 13 to 15 years from 2007 to 2011. Additionally, despite a decline in any tobacco use among the nation’s adults as reported in the Uganda Demographic and Health Survey 2006 and 2011[10, 11], our analysis indicates that the current use of tobacco of any kind has not changed among Ugandan youth during this period. Moreover, the percentage of current tobacco use of any type among Uganda students (17.3%) in this study was more than two-fold higher than the percentage among adults (7.9%) observed in previous research [12]. These statistics are concerning from a public health standpoint, as they may be an indication that the future prevalence of tobacco use among adult Ugandans could increase considerably. The fact that there was no change in current cigarette smoking and current use of other types of smoked tobacco between 2007 and 2011 could be due, in part, to the absence of MPOWER interventions, as outlined in the FCTC. Until July 2015, when Uganda enacted an FCTC compliant law, the only tobacco control related intervention was the smoke-free policy, which was not FCTC-compliant because important provisions such as 100% smoke free public places were not included in the law at the time [13]. These findings underscore the importance of implementing the evidence based interventions outlined in MPOWER, which have been shown to effectively prevent tobacco use among youth. In the present study, the use of non-cigarette forms of tobacco was found to be prevalent among youth, and at similar levels in both study periods. The use of both smoked and smokeless non-cigarette tobacco products is gaining popularity in many parts of the world, including many low- and middle-income countries [14]. Non-cigarette tobacco products are often less expensive than manufactured cigarettes and may be viewed by some as a safer alternative to smoking cigarettes [15, 16]. Among youth, the use of non-cigarette tobacco products, such as smokeless tobacco, is a concealable behavior that can occur unnoticed in school because it does not produce a noticeable smoke [17]. However, the use of non-cigarette tobacco products can result in nicotine addiction and consumption of other disease-causing chemicals, a particular concern for tobacco products that contain nicotine [16]. Accordingly, it is important for tobacco prevention and control efforts in Uganda to target all forms of tobacco use among young people, irrespective of whether it is combustible, non-combustible, or electronic.

This study found that exposure to both direct and indirect advertising, such as seeing tobacco advertisements in print media, being offered a free cigarette by a tobacco industry representative, and possessing an item with a tobacco logo did not change during 2007-2011. Article 13 of the WHO FCTC requires countries to prohibit tobacco advertising, promotion and sponsorship [18]. Advertising and promotion to youth generate an impression that the products are appropriate for both sexes, that they are stylish and fashionable, and that a young person’s peers will approve of tobacco use. Advertising is known to increase the consumption of tobacco products [19], which in turn increases disease burden and death [20]. In 1995, the Government of Uganda prohibited the advertisement of tobacco products on state media, which included state owned TV, radio and newspapers [21, 22].

This regulation has subsequently reduced direct advertising on billboards and other media that may not be necessarily state owned. However, advertising and promotion are still observed, especially at the point of sale [23]. Currently, there is not a comprehensive tobacco control law that includes provisions establishing restrictions on tobacco advertising, promotion, and sponsorship.

In the study, over 70% of students had been taught about the dangers and health effects of smoking tobacco during the past year. This fairly high level of exposure to health education is consistent with the fact that tobacco use is included in the normal curriculum for primary and secondary schools. In 2008, the Ministry of Health established a dedicated position for a tobacco control staff person to coordinate education, communication, training and public awareness on tobacco control activities and messages [22]. The focal person continuously engages government ministries, civil society organizations, other stakeholders, and the general public with information to adequately address current knowledge gaps on tobacco control. Additionally, the smoking behavior of parents and friends has been shown to be associated with youth tobacco use, indicating that a youth’s social environment can play a role in the initiation and continuation of smoking [24]. These considerations could be taken into account when designing or modifying behavior change interventions for students in Uganda.

### Limitations

The study is subject to at least four limitations. First, the sample was limited to school-going youths only, and therefore, may not be generalizable for all youth in Uganda. Second, students’ responses to the question about billboard advertisements could have been confused with the point of sale. In Uganda, an effective ministerial directive has prohibited tobacco billboard advertisements since 2005, and thus, few billboards with tobacco advertisements exist in the country. Third, a potential information bias from the self-administered nature of the questionnaire could lead to underreporting due to social desirability bias; however, reliability and validity measures that have been carried out on the GYTS data collection procedures indicate that the data are of acceptable quality [25]. Finally, due to the cross-sectional nature of the study, causality could not be established.

## Conclusion

While the prevalence of tobacco use among Uganda adults is decreasing according to the Uganda Demographic and Health Surveys, this study shows that the prevalence of current tobacco use among school going youth aged 13-15 years did not change during 2007-2011. Moreover the prevalence of tobacco use among youth is higher than adults. This has the potential to result in higher tobacco use prevalence in the next adult generation, which will result in increased premature deaths, morbidity and costs attributable to tobacco use. The newly signed WHO FCTC-compliant law on tobacco control in Uganda can guide comprehensive tobacco control programs countrywide, including complete prohibitions on tobacco advertising, promotion, sponsorship and comprehensive smoke free policies in public places. Among youth, the GYTS can effectively serve as a tool to evaluate the performance of tobacco control programs against MPOWER measures, at both national and local levels, as well as to monitor the implementation and compliance with the WHO FCTC. Regular implementation of these surveys and the proactive utilization of their results are paramount to inform and implement interventions, as well as to monitor progress towards FCTC compliance.

### What is known about this topic

- Tobacco use is dangerous in all its forms and its use among the youth is higher than that of adults in Uganda;
- The tobacco epidemic must be tackled through the enactment of policies that are premised in the domestication of the WHO Framework Convention of Tobacco Control.

### What this study adds

- This study highlights that there were no changes in the tobacco use prevalence of the youth despite several interventions by various partners and warns of potentially higher tobacco use prevalence in the next adult generation, which will result in increased premature deaths, morbidity and costs attributable to tobacco use;
- This study clearly highlights the urgency for Uganda to domesticate and implement a WHO Framework Convention of Tobacco Control complaint law to cut the tobacco epidemic.
